# Methods of quantifying a mass mortality event in freshwater wildlife within the river ecosystem

**DOI:** 10.1016/j.mex.2024.102567

**Published:** 2024-01-11

**Authors:** Dominik Marchowski, Agnieszka Szlauer-Łukaszewska, Łukasz Ławicki, Jacek Engel, Ewa Drewniak, Karol Ciężak

**Affiliations:** a Ornithological Station, Museum and Institute of Zoology of the Polish Academy of Sciences, ul. Nadwiślańska 108, Gdańsk 80-680, Poland; b Institute of Marine and Environmental Sciences, Szczecin University, Szczecin, Poland; c Eco-Expert, Szczecin, Poland; d Greenmind Foundation, Warszawa, Poland; e Save the Rivers Coalition, Poland; f Naturalist Club, Owczary, Poland; g The Society for Earth, Oświęcim, Poland

**Keywords:** Bivalvia, Gastropoda, Fish, Prymnesium parvum, Golden algae, Ecosystem collapse, Ecosystem Imbalance, River pollution, Ecological disaster, Invasive species, Quantifying a mass mortality events in freshwater wildlife

## Abstract

This study introduces a comprehensive method for quantifying mass mortality events in freshwater wildlife, exemplified by the ecological disaster in the Odra River in 2022. Our approach integrates field observations, statistical analysis, and ecological assessment to measure the impact of such events on various aquatic species. Key steps include systematic counting of deceased organisms, assessing population declines, and evaluating the ecological repercussions of invasive species. Utilizing the R programming language, we developed a framework that is adaptable to similar ecological crises in different aquatic environments. This methodology facilitates a detailed understanding of the scale and implications of mass mortality events, thereby contributing to effective environmental management and conservation efforts.

• The analysis and modeling methods of the disaster are presented in the R programming language.
• Exclusively open-source software was used for the analysis.
• The analysis includes detailed data on the disaster's impact on various species.

## Graphical abstract

**Figure:**
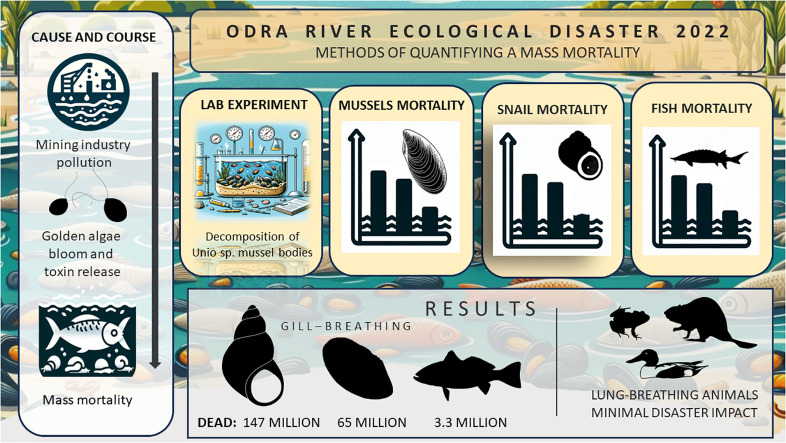

Specification table

**Table:** 

Subject area:	Environmental and Biological science
More specific subject area:	River ecosystem
Name of the method:	Quantifying a mass mortality events in freshwater wildlife
Name and reference of original method:	Szlauer-Łukaszewska, A., Ławicki, Ł., Engel, J., Drewniak, E., Ciężak, K., & Marchowski, D. 2024. Quantifying a mass mortality event in freshwater wildlife within the Lower Odra River: Insights from a large European river. Science of The Total Environment, 907, 167,898. 10.1016/j.scitotenv.2023.167898.
Resource availability:	Marchowski, Dominik; Szlauer-Łukaszewska, Agniszka; Ławicki, Łukasz; Engel, Jacek; Drewniak, Ewa; Ciężak, Karol (2023), “Quantifying a mass mortality event in freshwater wildlife within the Lower Odra River: Insights from a large European river. Data”, Mendeley Data, V1, doi:10.17632/hc63wkjtgr.1

## Background

In the summer of 2022, the Odra River in Europe experienced a severe ecological crisis, resulting in an extraordinary wave of deaths among fish, bivalves, and aquatic snails. This calamity was linked to the release of toxins by the golden algae *Prymnesium parvum*. The death toll of Unionidae mussels in the area Lower Odra River Valley reached approximately 65 million, a staggering 88% reduction in their numbers. The native mussel species, *Anodonta anatina*, encountered the most drastic decline of 95%, while the invasive *Sinanodonta woodiana* saw a 15% decrease. Moreover, at least 147 million deceased aquatic snails, mainly *Viviparus viviparus*, were discovered, suggesting an 85% drop in their population. Around 3.3 million fish, primarily ruffe *Gymnocephalus cernua*, bream *Abramis bram),* and perch *Perca fluviatilis*, were found dead along the lower Odra, totaling a biomass loss of 1025 tons. The overall fish mortality along the 560 km affected stretch of the river was estimated at 1650 tons, indicating a 60% decrease from the pre-disaster levels. The rapid decline of the river's ecosystem highlights the urgent need for additional research on its resilience and potential for recovery [1], [2], [3].

## Methods details

This section delineates the comprehensive methodology developed to quantify and analyze mass mortality events in freshwater ecosystems, as demonstrated by the ecological disaster in the Odra River in 2022. Our approach is designed to be universally applicable to similar ecological crises, providing a robust framework for assessing the scale and impact of such events on various aquatic species.

Central to our method is the use of the R programming language [4], which enables precise and adaptable statistical analysis. This choice of software facilitates the replication and customization of our methods in different ecological contexts. The detailed R scripts and methodologies have been made publicly available in the Zenodo repository [2], ensuring transparency and ease of access for other researchers.

To illustrate the practical application of our methods, we have provided raw data compatible with our R scripts, as used in the main article, in the Mendeley repository [3]. This allows for a clear demonstration of how our methodology can be applied to real-world data, encouraging its use in diverse ecological scenarios.

## Decomposition of *Unio* sp. mussel bodies – laboratory experiment

### Methodology

This methodology presents a standardized procedure for examining the decomposition of *Unio* sp. mussel bodies in a laboratory setting. The aim is to simulate various natural aquatic environments for a comprehensive understanding of the decomposition process in freshwater mussels, a key factor in ecological disaster assessments.

The mussels should be humanely euthanized following the legal guidelines of the country in which the research is conducted. In the experimental setup, the mussels are placed in an artificial water basin, with conditions tailored to reflect their natural habitat. This involves adjusting water temperature and sediment type (including sandy, muddy, or rocky substrates) to mirror the natural environment pertinent to the disaster being studied. Such a controlled setup facilitates detailed observations of the decomposition process under ecologically relevant conditions.

### Validation

For the validation of this method in the context of the Odra River disaster, the experiment was conducted in Poland, adhering to humane euthanasia practices as per Polish regulations [5,6]. The artificial basin replicated the conditions of the Odra River during the disaster, with sandy sediments to simulate the riverbed and water temperature maintained at around 23 °C, aligning with the environmental conditions reported during the disaster [7,8]. Applying this method to the specific case of the Odra River provided valuable insights into the decomposition behavior of *Unio pictorum* and *Unio tumidus* mussels under similar environmental conditions, thus validating the method's effectiveness and adaptability to real-world scenarios.

## Comparison of the number of mollusks before and after the catastrophe

### Methodology

This method outlines a standardized approach for comparing mollusk populations (mussels and water snails) before and after an ecological disaster. Sampling should be conducted at designated points to assess changes in populations over time.

Sampling should incorporate two techniques: diving and net collection. Divers should be equipped with flashlights and tasked with collecting mussels directly from the riverbed. Concurrently, a net with a standardized hydrobiological mesh should be used for collecting both mussels and snails. The specifications of the mesh, such as inlet width and mesh size, must be consistent across all sampling efforts. Collected samples need to be averaged, with densities converted to an area of 1 m² for standardization and expressed as the number of individuals per m².

For statistical analysis, data normality must first be assessed using histograms, Q-Q plots, and the Shapiro-Wilk test. Depending on the data distribution, either a one-way ANOVA (for normally distributed data) or a non-parametric Kruskal-Wallis test (for non-normally distributed data) should be used to compare population densities, percentages, and frequencies between different time points.

### Validation

This method was validated in the context of the Odra River ecological disaster. Samples of mollusks were collected at 13 points from September to November in both 2017 and 2022 [1]. The surveyed areas were 46 m² in 2017 and 51 m² in 2022. The application of the outlined statistical tests to these samples demonstrated significant differences in mollusk populations, thereby validating the method's effectiveness in quantifying the impact of the disaster. The raw data, as well as the R programming language codes, intermediate, and final results, are available in the Mendeley [4] and Zenodo [3] repositories.

## Estimating the number of dead mussels flowing in the river current

### Methodology

This method outlines how to estimate the number of dead mussels from the Unionidae family visible in a river's mainstream, using the point transect approach principles [9]. The method involves observing dead mussels on the water surface as they pass through a designated line perpendicular to the shoreline. The strip transects sampling method [10] should be employed for this observation.

Data collected from these observations must be corrected for detectability, employing an average detection rate. The formula for the average number of individuals per minute, corrected for the detection rate, is as follows:

$$\bar{x}=[(\frac{1}{n}\sum_{i=1}^{n}x_{i}/\omega ]/5)$$

Where x is the number of individuals in the sample, n is the sample size, and ω is the average detection rate, assumed to be arbitrary. The final count should be extrapolated to a daily estimate by multiplying by the number of minutes in a day. Additionally, 95% confidence intervals should be calculated for the estimated values.

By utilizing data on the river's current speed, the method allows for the estimation of the potential distance that mussels may have traveled from their origin to the survey point.

### Validation

This method was validated during the ecological disaster on the Odra River. The count was performed from a riverbank point in Krajnik Dolny on August 17, 2022, using five-minute sessions at two-hour intervals [1]. The resulting data were corrected for detectebility [11]. An average detection rate of 0.6, as reported by Ronconi and Burger [12], was used to correct the data for detectability. The State Water Holding Polish Waters provided data on the average water flow (https://www.pgw.wody.gov.pl), enabling the estimation of the original section of the river from which the mussels originated. This validation demonstrated the method's practical applicability and effectiveness in a real-world scenario. The raw data and R programming language codes, along with intermediate and final results, are available in the Mendeley [4] and Zenodo [3] repositories.

## Estimation of the number of dead mussels on the riparian zone of the river

### Methodology

This method details how to estimate the number of dead mussels washed ashore along riverbanks. The approach involves conducting surveys on randomly located ten-meter transects along a designated river section. Generalized additive modeling (GAM) should be used to explore the relationship between the number of mussels and two predictor variables: site (transect location) and river kilometer. This allows for the analysis of non-linear relationships between these variables [13].

Subsequently, the Generalized Estimating Equations (GEE) model should be employed to calculate the average rate of change in mussel numbers over successive river sections [14]. The GEE approach can also be used to predict the number of mussels across an entire river section. These predicted values should be then averaged and extrapolated over the length of the studied river section using the formula:

1. Początek formularza

$$\bar{x}=\frac{1}{n}\sum_{i=1}^{n}x_{i}$$

Where *x_1_, x_2_,…x_n_* are samples (number of mussels found on 10 m transects) and predicted values of the number of individuals in subsequent sections.

$$\hat{x}=2L\bar{x}$$

Where $\hat{x}$ is the result of extrapolation and *L* is the length of the river.

### Validation

**Fig. 1:**
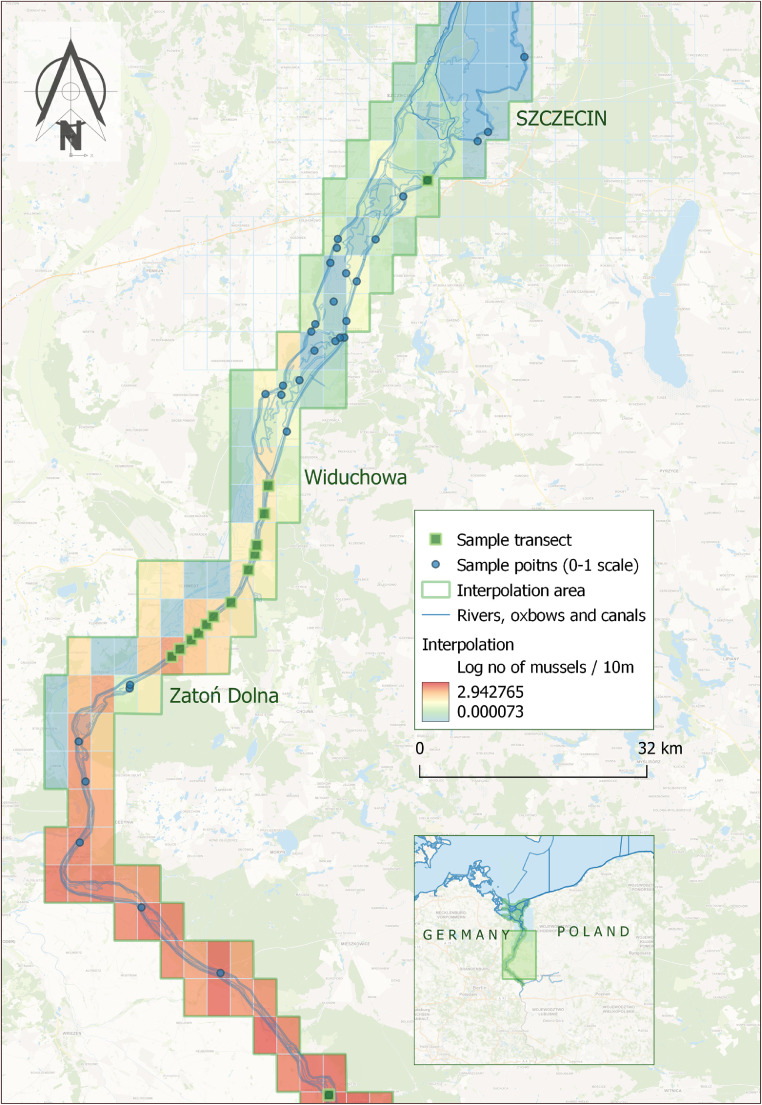
Interpolation of mussel mortality scale in the Lower Odra Valley, the Odra Estuary in summer 2022.

This methodology was validated through a survey conducted in the middle part of the lower Odra River, from Zatoń Dolna to Widuchowa, and additional points from August 18–26, 2022 [1]. The survey included 12 transects and 56 random points along the riverbank. The GAM and GEE models were used to predict mussel numbers, and the results were averaged and extrapolated over a 109 km section from Kaleńsko to Szczecin. The Western Odra section of 26 km was also included in the analysis (Fig. 1).

The model's positive values were log-transformed and interpolated onto a map using the Inverse Distance Weighing (IDW) method in QGIS ver. 3.20.3 [15,16], illustrating the spatial distribution and density of mussels washed up on the banks (Fig. 1).

The raw data required to perform the analyses are located in the Mendeley repository [4], while the codes in the R programming language, along with intermediate results (e.g., analysis of data normality), and the final results are available in the Zenodo repository [3].

## Estimating the scale of total mortality of mussels from the Unionidae family

### Methodology

To estimate the total mortality of mussels from the Unionidae family in a river ecosystem, one should consider the typical habitat of live mussels and the specific environmental conditions of the river. It is assumed that mussels generally occupy a 20-meter strip on both sides of the riverbed, particularly in areas like the bottom of the coastal zone and spaces between spur dikes, where the current is slower [5].

The methodology involves calculating the population abundance before and after a disaster by considering the mussel density, the length of the assessed river section, and the width of the occupied bottom. The river's length should be measured from relevant points, and the total length is to be multiplied by the width of the suitable habitat strip on both sides. This area is then further multiplied by the average mussel densities to estimate the total population.

### Validation

This method was applied to the Odra River [5]. The length of the Odra River was determined from the mouth of the Warta River at kilometer 617.6 to the mouth of the Regalica River at kilometer 741.6, resulting in a total length of 124 km. An additional 26 km was included for the Western Odra, totaling 150 km. The length of the river was multiplied by the width of the suitable habitat strip. This calculated area was then multiplied by the average mussel densities, estimated at 12.6 individuals per square meter before the disaster and 1.6 individuals per square meter afterward, as obtained from the study detailed in the previous section. By subtracting the pre-disaster abundance estimates from the post-disaster figures, the total scale of mussel mortality due to the event was accurately calculated.

The raw data and R programming language codes, including intermediate and final results, are available in the Mendeley [4] and Zenodo [3] repositories for this analysis.

## Estimating the scale of water snail mortality

### Methodology

To estimate water snail mortality in river ecosystems, random photographs of riverbanks should be taken to document dead animals found washed ashore. These photos must capture a representative sample of the animal groups present, typically including fish, mussels, and water snails. For qualitative and quantitative analysis, the photos should be annotated in a manner similar to the preparation for neural network training in machine learning [17,18].

A square grid should be superimposed on each photo to facilitate counting. Care must be taken with photos captured with perspective to ensure accurate object recognition. In cases where recognition is inadequate, a demarcation line should be drawn, and objects beyond this line should not be included in the count. The average number of snails in the photos can then be compared to the number of mussels, and using the estimated number of mussels washed ashore, the number of dead water snails can be calculated proportionally.

### Validation

**Fig. 2:**
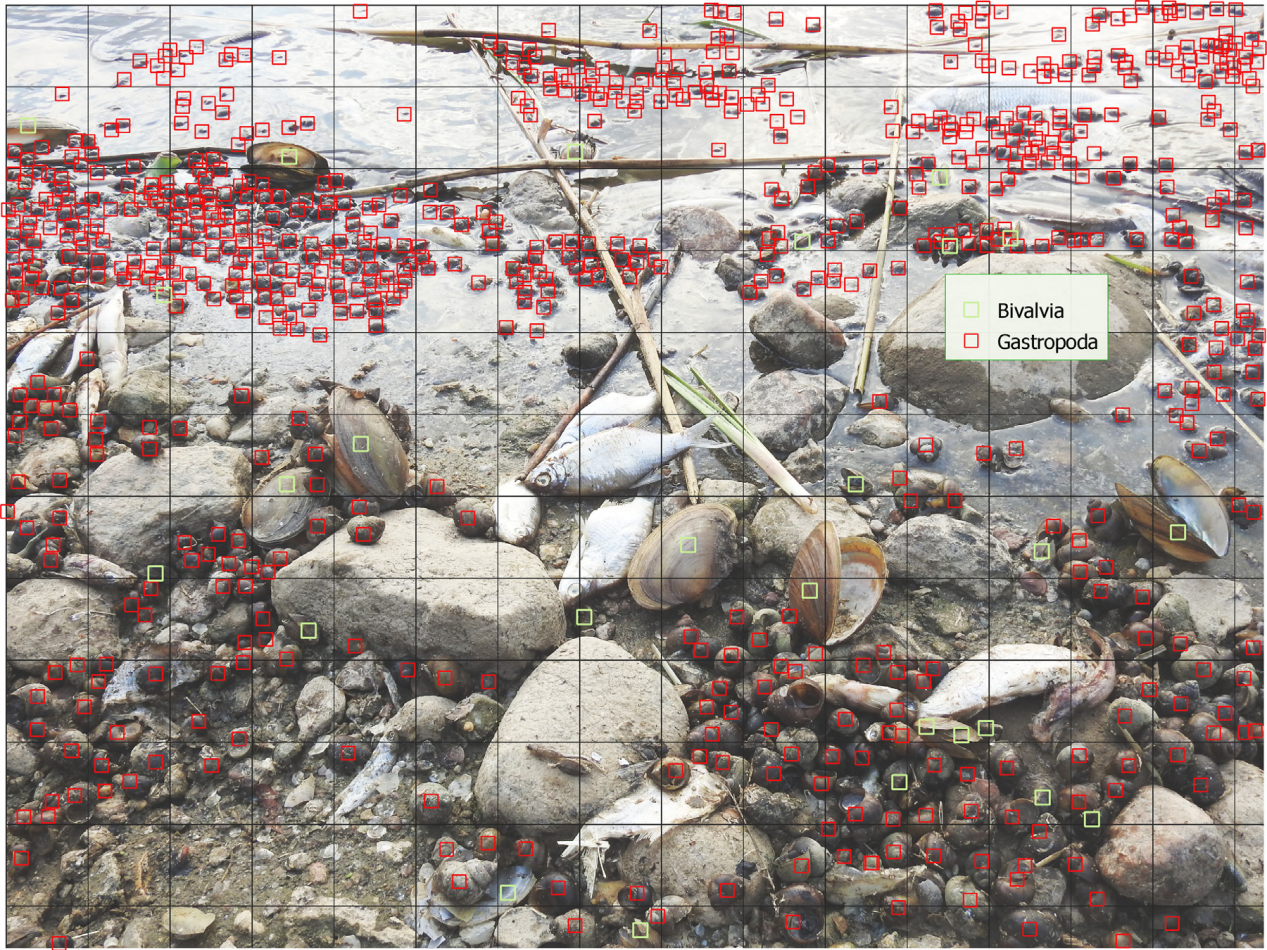
An exemplary annotated photo on the basis of which the proportion of dead snails in relation to mussels was assessed. Red demarcation line – objects beyond this line were not included in the count. (source: Fig. 10 from Szlauer- Łukaszewska et al. [1]).

This method was applied in the Lower Odra Valley from Kostrzyn to Szczecin. Thirteen photos were annotated to analyze the species of animals washed ashore, mainly consisting of fish, mussels, and water snails. The annotation process followed guidelines similar to those used in machine learning [17,18], and a square grid was used to aid in counting (Fig. 2). In cases of inadequate recognition, a red demarcation line was drawn to exclude objects beyond it from the count. Based on these annotations, the proportion of dead snails in relation to mussels was assessed [1].

The raw data, R programming codes, intermediate results, and the final explanation of the results are available in the Mendeley [4] and Zenodo [3] repositories.

## Evaluation of vertebrate mortality

### Methodologyffig

To evaluate vertebrate mortality in a river ecosystem, a comprehensive survey should be conducted along randomly selected transects in the river valley. Transects, each 10 m long, should be chosen to cover a representative section of the river. Sampling should be carried out in all existing habitat types along the riverbank, such as stony, gravel, reed-covered, and sandy banks.

Within each selected section, a thorough search and count of all common vertebrates, including lampreys, fish, amphibians, birds, and mammals, should be conducted. Species should be classified and tallied at the species level or higher taxonomic units.

The estimated number of vertebrates should be extrapolated to the entire representative length of the river. This extrapolation in the case of fish should also convert the number of individuals into weight for comparison with other publications and reports [7,19]. Data on the average size of individual fish species can be obtained from measurements at sample points along the river. A commonly used converter with species-specific conversion factors should be employed for estimating the weight of the fish [20].

Statistical analysis, including the Kruskal-Wallis test and Generalized Additive Models (GAM) [13], should be used to examine relationships between fish abundance and location, as well as other influencing factors like shore habitat.

### Validation

In the middle section of the Lower Odra Valley, from Zatoń Dolna to Widuchowa, 43 transects were selected over approximately 20 km (Fig. 1). Comprehensive searches were conducted from August 18–26, 2022, for vertebrates affected by the disaster [1]. The Kruskal-Wallis test and GAM analysis were employed to evaluate fish abundance and its relationship with location and shore habitat [13,20].

The length of the Odra River from Lipki to Szczecin (561 km) and the lower section of the Odra River (150 km) were considered for the extrapolation of fish abundance. The results were compared with the published estimation of fish mortality due to the disaster [19] and with estimations for the lower Odra.

Published reports and data from the disaster period were reviewed, with only those methodologies that could be verified being utilized. Searches were conducted in scientific databases and general search engines.

The raw data, R programming codes, and the results of these analyses are available in the Mendeley [4] and Zenodo [3] repositories.

## Determination of taxa affected by the disaster, qualitative analysis

### Methodology

To determine the taxa affected by an ecological disaster, a comprehensive qualitative analysis should be conducted. This involves a series of systematic efforts to identify the impact on various forms of aquatic life, including mussels, snails, fish, and other wildlife species. The methodology includes several key components:

- Systematic counting of deceased mussels found floating in the river.
- Tallying deceased mussels stranded on riverbanks.
- Analyzing photographs taken during fieldwork for taxonomic identification.
- Collecting benthic samples from the riverbed for examination.
- Conducting detailed site inspections within the study area.

The taxonomic identification process should involve the classification of species or other relevant taxonomic levels based on photographic images and physical samples. This should cover a range of species, from mussels and snails to fish and other wildlife.

### Validation

In the Lower Odra Valley, from Kostrzyn to Szczecin, considerable efforts were made to identify the species impacted by the disaster. Photographic documentation and systematic fieldwork revealed a variety of deceased mollusks, including *Anodonta anatina, Sinanodonta woodiana, Unio pictorum, Unio tumidus*, and several others. No deceased waterbirds or amphibians were encountered during the study, although live *Pelophylax* frogs and two instances of dead water shrew *Neomys fodiens* were observed.

The initial phases of the study focused on deceased mollusks in the river and on its banks, while subsequent phases expanded to include a broader range of species. The detailed analysis of photographs and site inspections provided a comprehensive assessment of the species affected by the disaster.

## Conclusion

This study has developed a comprehensive and adaptable methodology for quantifying the impact of ecological disasters on aquatic wildlife, as exemplified by the disaster in the Lower Odra River. Our approach, integrating field observations, statistical analysis, and ecological assessment, is designed to be universally applicable to various aquatic environments and ecological crises. The methods outlined offer a robust framework for accurately assessing mass mortality events, encompassing various species from mussels and snails to fish and other vertebrates. The validation of these methods in the context of the Odra River disaster underscores their practical utility and effectiveness in real-world scenarios. This methodology contributes significantly to environmental research, offering a systematic approach to understanding and managing the consequences of ecological disasters, thereby enhancing our capabilities for effective environmental conservation and management in the face of future challenges.

## Ethics statement

The authors adhere to the ethical guidelines of the journal. While this research involved activities with wild animals, all procedures were in strict compliance with national law and European Union regulations. No humans or data from social media were involved in this study.

## Addictional information, data availability

*All raw data needed for the analyzes carried out in this article are available at:* Marchowski, Dominik; Szlauer-Łukaszewska, Agnieszka; Ławicki, Łukasz; Engel, Jacek; Drewniak, Ewa; Ciężak, Karol (2023), “Quantifying a mass mortality event in freshwater wildlife within the Lower Odra River: Data”, Mendeley Data, V1, 10.17632/985dh5r9cs.1

*All codes and explanation needed for the analysis can be found at:*Marchowski, D. (2023). R codes for quantifying a mass mortality event in freshwater wildlife within the Lower Odra River. In: Science of the Total Enironment (T. 907, Number 1, s. 167,898). Zenodo. 10.5281/zenodo.10066315

## CRediT authorship contribution statement

**Dominik Marchowski:** Conceptualization, Data curation, Formal analysis, Funding acquisition, Investigation, Methodology, Project administration, Supervision, Validation, Visualization, Writing – original draft, Writing – review & editing. **Agnieszka Szlauer-Łukaszewska:** Conceptualization, Data curation, Formal analysis, Funding acquisition, Investigation, Methodology, Project administration, Validation, Visualization, Writing – original draft, Writing – review & editing. **Łukasz Ławicki:** Conceptualization, Data curation, Investigation, Methodology, Validation, Writing – review & editing. **Jacek Engel:** Conceptualization, Data curation, Investigation, Methodology, Validation, Writing – review & editing. **Ewa Drewniak:** Conceptualization, Data curation, Investigation, Methodology, Validation, Writing – review & editing. **Karol Ciężak:** Conceptualization, Data curation, Investigation, Methodology, Validation, Writing – review & editing.

## Declaration of competing interest

The authors declare that they have no known competing financial interests or personal relationships that could have appeared to influence the work reported.

## Data Availability

There are links to raw data and codes in the text.
